# Higher Education as a Driver for the Humanization of Pediatric Pain Care (HUPEDCARE): Protocol of a Multicenter Study

**DOI:** 10.3390/ejihpe16040056

**Published:** 2026-04-20

**Authors:** Sagrario Gómez-Cantarino, Henrique Ciabotti Elias, Miriam Hermida-Mota, Pablo Pando Cerra, Deisa Salyse dos Reis Cabral Semedo, Ana Suzete Baessa Moniz, Sonsoles Hernández-Iglesias, Ana Maria Aguiar Frias, Tuğba Erdem, Maria da Conceição Fernandes Santiago, Inmaculada García-Valdivieso, Amelia Marina Morillas Bulnes, Jahit Sacarlal, Renata Karina Reis

**Affiliations:** 1 Department of Physiotherapy and Nursing, University of Castilla-La Mancha, 45004 Toledo, Spain; sagrario.gomez@uclm.es (S.G.-C.); inmaculada.garciavaldivieso@alu.uclm.es (I.G.-V.); 2 Health Sciences Research Unit: Nursing (UICISA: E), Coimbra Nursing School (ESEnfC), 3046-851 Coimbra, Portugal; 3 Ribeirão Preto School of Nursing, University of São Paulo, Campus Universitário, Ribeirão Preto 14040-900, SP, Brazil; henriqueciabottielias@usp.br (H.C.E.); rkreis@eerp.usp.br (R.K.R.); 4 Department of Construction and Manufacturing Engineering, University of Oviedo, 33203 Gijon, Spain; pandopablo@uniovi.es; 5 Faculty of Science and Technology, University of Cape Verde, Praia CP 279, Cape Verde; deisa.semedo@docente.unicv.edu.cv; 6 Science Nature Life and Environment Unit (UCNVA), Jean Piaget University of Cape Verde, Praia CP 11, Cape Verde; azm@cv.unipiaget.org; 7 Faculty of Health Sciences, Francisco de Vitoria University Foundation, Pozuelo de Alarcón, 28223 Madrid, Spain; s.hernandez@ufv.es; 8 San João de Deus School of Nursing, Comprehensive Health Research Center (CHRC), University of Évora, 7000-811 Evora, Portugal; anafrias@uevora.pt; 9 School of Nursing, Koç University, Zeytinburnu, Istambul 34010, Türkiye; tyeni@ku.edu.tr; 10 School of Health, RISE-Health, Santarém Polytechnic University, 2001-904 Santarem, Portugal; mconceicao.santiago@essaude.ipsantarem.pt; 11 Nursing Faculty, National University of Trujillo, Trujillo 13011, Peru; amorillas@unitru.edu.pe; 12 Department of Microbiology, Faculty of Medicine, Eduardo Mondlane University, Maputo CP 257, Mozambique; jahit.sacarlal@uem.mz

**Keywords:** higher education, innovation, pediatric pain, humanization of pain, educational innovation, educational technology in healthcare

## Abstract

Pediatric pain remains a highly prevalent and under-addressed health problem worldwide, largely due to educational gaps, limited humanization of care, and insufficient integration of digital and pedagogical innovations in higher education, and the purpose of this study is to describe and implement an international, higher education–driven model to improve training in humanized pediatric pain management. This multicenter mixed-methods study involves 15 universities from Europe, Africa, and Latin America and includes the development and cross-cultural validation of the HUPEDCARE-Q questionnaire to identify knowledge gaps, the design of an open-access, multilingual digital learning platform (PEDCARE) that integrates learning management and social networking functions, and the implementation of capacity-building workshops based on a training-the-trainers model for students, educators, health professionals, and families. The expected outcomes of the project include the establishment of a standardized instrument for assessing educational needs, the creation of a scalable digital educational environment, and the feasibility of international academic collaboration to strengthen competencies in pediatric pain care. The study suggests that higher education, combined with digital transformation and culturally sensitive approaches, may support the humanization of pediatric pain management and address educational and health inequities, although further research is needed to confirm these potential impacts.

## 1. Introduction

Research on pain and its effects is a field of research of international interest. According to the International Association for the Study of Pain (IASP), pain is defined as “an unpleasant sensory and emotional experience associated with, or described in terms of, actual or potential tissue damage” (Raja et al., 2020). Pain has a subjective component that is delineated by previous life experience (Cuyul Vasquez & Araya-Quintanilla, 2019; Cáceres-Matos et al., 2019). Pain is classified according to its duration, pathogenesis, course and intensity (Wscieklica et al., 2024). According to the World Health Organization (WHO), a distinction is made between acute or chronic (primary or secondary) pain (WHO, 2024). Depending on its pathogenesis, it can be neuropathic, somatic/visceral nociceptive or mixed, and in terms of its course, mild, moderate or severe, according to the validated pain assessment scales used, depending on age (Leyva Carmona et al., 2019).

Chronic pain affects 1 in 5 children and adolescents and is a common health problem today. Headache and musculoskeletal pain are prevalent in the general pediatric population, although gender differences exist. Generalized pain, headache and back pain are more prevalent in girls (Chambers et al., 2024; Checa-Peñalver et al., 2024). Despite the existence of several pain assessment scales, healthcare workers face barriers to pain management such as lack of unification of pain recording, problems in clinical information exchange, poor baseline information on treatment and on how to implement non- pharmacological techniques (Leyva Carmona et al., 2019).

Age, cognitive level, presence of disabilities and type of pain should be considered when assessing pediatric pain (Teisseyre et al., 2018). Research on chronic pain is scarce compared to acute pain (Eijlers et al., 2019; Suleiman-Martos et al., 2022; Wren et al., 2019), so more clinical trials and longitudinal studies should be conducted to ensure the structure of evidence-based guidelines and training to achieve complete pain relief in the pediatric patient following a behavioral, physical and non-pharmacological approach to prevent progression to chronicity (Lebel & Schuster, 2024). In this context, the humanization of care can be defined as an approach that recognizes each patient’s individuality, dignity, and emotional needs, integrating empathetic, relational, and ethical principles into clinical practice, in line with Watson’s Theory of Human Caring (Contrera & Rodríguez, 2022).

Non-pharmacological interventions have been shown to have multiple benefits in the management of neonatal pain (Mangat et al., 2018; Aguilar-Cordero et al., 2015). The need to implement them within interdisciplinary care planning is a requirement to ensure and promote the humanization of care (Collins, 2023). The adoption of a patient-centered approach, where all spheres of the patient (bio-psycho-social) are valued, is relevant in the management of such a vulnerable population (Dudley et al., 2015). This approach creates a positive impact on the infant’s cognitive development and is useful for promoting emotional well-being and improving the relationship between healthcare staff and families (Reyes-Téllez et al., 2024; López Ramiro et al., 2023).

Increasing the level of knowledge on the humanization of pediatric pain management is possible through its implementation in Higher Education (HE) training, including specialized training in pediatrics in healthcare careers, which is key to optimizing the quality of care provided to professionals (Mozo del Castillo et al., 2022; Notejane et al., 2016). Acting from HE is the starting point to promote learning about comprehensive care, including non-pharmacological techniques for pediatric pain, and to train them to deal with situations that require complex pain management in this population. The synergy between these actions and the implementation of new Information and Communication Technologies (ICT) that facilitate the acquisition and accessibility of evidence-based knowledge contributes to establishing an effective and ethical orientation in healthcare for pediatric patients. In this way, competencies and practical tools are strengthened to enable healthcare professionals to broaden the basis of their training (Trottier et al., 2022; Warren, 2017).

In alignment with Sustainable Development Goal 3 (SDG 3): Good Health and Well-Being, which advocates for ensuring healthy lives and promoting well-being for all at all ages, the inclusion of countries such as Mozambique, Cabo Verde, Peru, and Brazil becomes a strategic priority (Sanhueza et al., 2022). In addition to a clear language (13 of 15 parts of the project in Spanish and/or Portuguese), there are multiple historical and cultural connections between Europe, Africa and Latin America. Another important factor in this selection was the recent population ranking by the United Nations Population Fund (UNFPA) (United Nations Population Fund, 2025), which highlights that African countries occupy the top positions in terms of the proportion of children aged 0 to 14: 43.53% in Mozambique and 26.1% in Cabo Verde. South America also stands out in this regard, with 26.01% of the population in this age group in Peru and 20.27% in Brazil. Therefore, given the high prevalence of pain in the pediatric population, it is essential that educators, students, and healthcare professionals in these selected regions enhance their capacity to care for children, supported by the results generated through this project (Tabutin & Schoumaker, 2020).

In HE, both Mozambique and Cabo Verde maintain academic links with Portugal, which enables the development of a transnational dimension in pediatric pain management within the scope of this project (Augé, 2021; Almeida Aguiar, 2023). The historical processes of technological, economic, and cultural transformation underscore the necessity for Africa and Latin America not to be left behind in the current revolution centered around Information and Communication Technologies (ICT), a field to which this project is intrinsically connected (Astudillo Castro et al., 2018; Humanante-Ramos et al., 2019; Karsenti & Lira, 2011).

To address the global situation, the HUPEDCARE study is presented as an innovative and multidimensional response that integrates technology through the PEDCARE platform, conceived as a learning management system combined with a technical social network, together with the validation of the HUPEDCARE-Q questionnaire, constituting a pioneering advance toward the standardization and humanization of pediatric pain management across diverse contexts. Therefore, it is essential to strengthen current approaches to pediatric pain management through strategies that are both scientifically grounded and culturally sensitive. Accordingly, the objective of this project is to Implement transformative actions in institutions in Europe, Africa, and Latin America through training programs that integrate technological innovations.

## 2. Method

The research is exploratory and descriptive multicenter and multi-media study with a qualitative and quantitative approach (Ruiz Bolivar, 2008) that will be carried out over a period of three academic years from 1st October 2024 to 30th September 2027. It involves the participation of 15 partner universities from 3 different continents (Asia, Africa, and America), making it a multicenter study, in which institutions work together. The partner universities participating in this project have joined forces in a consortium and under contract to create the project “HE as a driver for the humanization of pediatric pain management” (HUPEDCARE), in the 2024 call for international projects on capacity building in HE by the European Education and Culture Executive Agency (EACEA), with the code ERASMUS-EDU-2024-CBHE-101177475. After being positively evaluated by peers and selected within the call, this project was funded by this European organization.

The HUPEDCARE project seeks to transform HE in Africa and Latin America using technology and academic cooperation to improve the humanization of pediatric pain care (Beleño Gallego et al., 2024). The main objective is to identify and implement transformative actions in institutions in Europe, Africa and Latin America that enable integration capacity building, academic cooperation between HE institutions and the transmission of knowledge to society, in relation to the humanization of pediatric pain care. This objective will be achieved by implementing technological solutions in HE to expand the tools and knowledge base of healthcare professionals. The specific objectives through which the main objective will be pursued are as follows:

(1) Validate a questionnaire to detect gaps in knowledge about pediatric pain management and non-pharmacological techniques for pain relief in pediatrics “Humanization of Pediatric Care in Pain Management with a Non-Pharmacological Approach (HUPEDCARE-Q)”.
(2) Generate applied knowledge on the humanization of pediatric pain management in institutions in Latin America, Africa, and Europe, using technological resources and training the trainers among students, healthcare professionals, and academic professionals.
(3) Create an open access, multi-device and multilingual web portal (PEDCARE Platform) that will be a learning management, monitoring and evaluation system (Learning Management System, or LMS) and a Social Networking Site (SNS) connecting experts in pediatrics and students.

**Figure 1:**
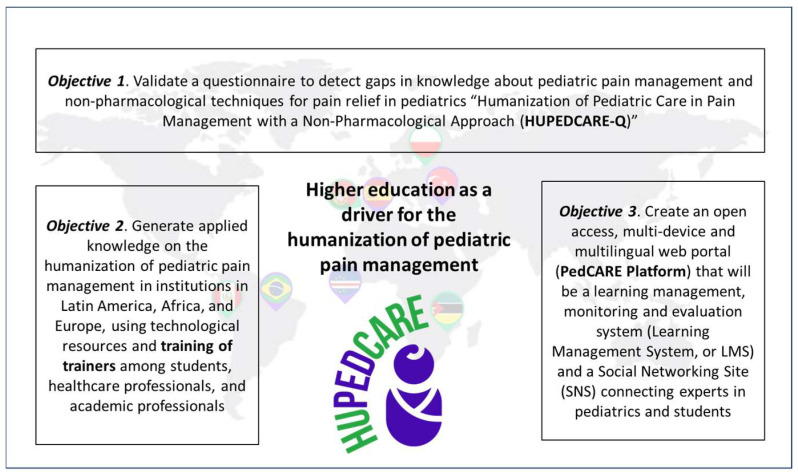
Three objectives of HUPEDCARE study. Source: authors’ own elaboration.

The main objective of HUPEDCARE will be achieved through the success of the three objectives described above and reflected in Figure 1.

**Figure 2:**
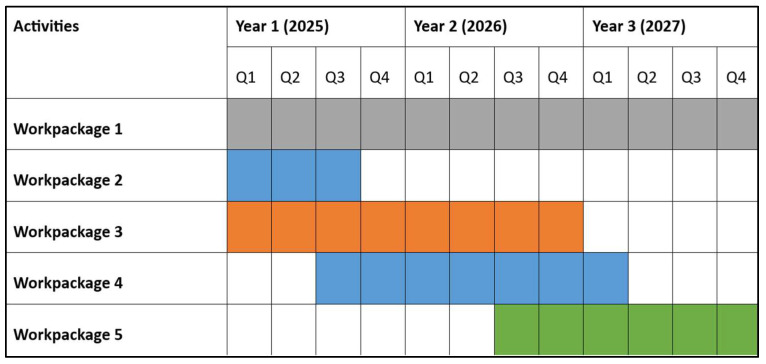
HUPEDCARE project timeline (Gantt diagram). Source: authors’ own elaboration. Q: quartile. The colors indicate the duration of each WP for quick identification.

The project is structured into work packages (WPs) designed to organize the project and its tasks efficiently, facilitating the achievement of objectives and promoting collaboration among the participating universities. Each WP is associated with numerous tasks and is led by a different university. In this way, the collaboration among all university partners ensures the attainment of the project’s four main outcomes. In addition, each of them will be developed within a specific time frame, as illustrated in Figure 2.

### 2.1. Ethical Approval and Data Protection

The protocol was approved by the Social Research Ethics Committee of the University Castilla-La Mancha (UCLM) in Toledo, Spain, under the code CEIS-2025-91937. The leaders of the 15 universities signed a confidentiality clause to protect the sensitive data of the project and guarantee its appropriate use. Also, it has been approved by the Clinical Research Ethics Committee (C.E.I.m) under number 1377 on 11/06/2025 by the Toledo University Hospital Complex, Castilla-La Mancha Health Service (SESCAM).

### 2.2. Workpackage 1: Management and Coordination of the Project

The University of Castilla-La Mancha will manage and coordinate the project and will be responsible for patenting the HUPEDCARE project brand for international identification. In addition, all universities will work and replicate the efforts on each campus and in each community. The project will grow and be enriched by the contributions made by all participants, both in the transnational telematic and face-to-face meetings that will be held periodically, and in the training activities and conferences that will be held on the humanization of pediatric pain care. Three committees (management, quality and conflict resolution) and a partners’ council will be formed for decision making. In this way, the aim is to achieve the greatest possible fulfillment of all the programmed activities to achieve success in the three specific objectives described thanks to the collaboration and commitment of all the partners.

### 2.3. Workpackage 2: Global Analysis on the Humanization of Pediatric Pain Care in HE: HUPEDCARE-Q Questionnaire and Knowledge Base for Training the Trainers

This analysis is necessary to visualize real needs and define the actions and measures that must be implemented to develop training and expand knowledge in a satisfactory manner. HUPEDCARE aims to improve training in the humanization of pediatric pain care in HE by analyzing the attitudes, beliefs and knowledge of healthcare professionals internationally. To this end, five universities will work together to (a) Develop and validate a questionnaire to determine the attitudes, beliefs, and basic training of healthcare professionals regarding pediatric pain and its non-pharmacological management; (b) Publish a report to disseminate the results and the baseline situation at the international level on this subject and identify its strengths and weaknesses; and (c) Generate the knowledge base for subsequent training the trainers on the humanization of pediatric pain care. It will include a quantitative study on the international situation and a qualitative study on university professors. 

It is necessary to develop a questionnaire to determine attitudes, beliefs, and basic knowledge of healthcare professionals from their foundational training. To this end, the questionnaire will be reviewed, modified and validated by a multidisciplinary group of experts. For the initial validation, a sample of 1120 healthcare professionals in Spain will be used to validate the questionnaire in Spanish. Following this successful validation, the questionnaire will consist of four sections. The first section of the questionnaire is designed to collect sociodemographic data from participants. The second section of the questionnaire focuses on exploring attitudes and beliefs about pediatric pain, and to this end, a review of the literature was conducted to compare the different positions and thoughts of healthcare professionals on this topic (Ung et al., 2016; Chow & Chan, 2015; Maixé & Miró, 2016; Baarslag et al., 2019). The third section considers the knowledge and training received in health related to pediatric pain, focusing on the professional’s understanding of pediatric pain and teaching methods in HE (Kusi Amponsah et al., 2020). The fourth and final section contains 10 items on best practices for addressing pediatric pain, presenting statements/denials about aspects of behavior, emotions, and cognitive aspects in pediatric pain responses as perceived and expressed by the child experiencing it during their development (J. A. Schreiber et al., 2014).

To evaluate the psychometric properties of the instrument, content validity, internal consistency, and factor structure will be assessed. Content validity will be examined through expert judgment using the Content Validity Coefficient (CVC) (Pedrosa et al., 2013). The underlying structure of the questionnaire will be explored using an exploratory factor analysis (EFA), with data adequacy verified by the Kaiser–Meyer–Olkin index and Bartlett’s test of sphericity (J. B. Schreiber, 2021; Pizarro Romero & Martínez Mora, 2020). Internal consistency will be assessed using Cronbach’s alpha for each questionnaire dimension (Oviedo & Campo-Arias, 2005).

This initial validation in Spanish will be carried out by the Spanish universities participating in the project, with the aim of conducting validation in a first language as the main coordinator of the project with the support of the other Spanish universities.

Beyond linguistic translation, cross-cultural validation will include procedures to ensure measurement equivalence across versions. This process will follow a forward and backward translation protocol to ensure semantic consistency between the original and target versions (Wild et al., 2005). These procedures will also involve expert review panels in each language to assess face and content validity, as well as pilot testing in the target populations to evaluate item clarity and cultural relevance (Beaton et al., 2000). Measurement invariance across language versions will be examined using factorial structure comparisons (Vandenberg & Lance, 2000), and reliability indices will be calculated for each version to confirm the consistency of the instrument across cultural contexts (Sousa & Rojjanasrirat, 2011).

Building upon this methodological framework, the questionnaire will be translated and validated in multiple languages—including Turkish, Brazilian Portuguese, Polish, English, Latin American Spanish, and regional variants of Portuguese—to ensure its applicability across diverse cultural and linguistic settings. Following this rigorous transcultural validation across the participating universities, the instrument will be launched internationally to facilitate a comprehensive global analysis of the situation (Alavi et al., 2025; Cruchinho et al., 2024).

To this end, an extensive list of contacts (universities, private or public institutions or associations, professional associations) will be drawn up in the most representative countries on all continents. The invitation to participate in the study will be sent via the official HUPEDCARE website. The questionnaire is intended to be sent to 257,954 people. The sample size has been calculated with the GRANMO software (v7.12) estimating, with a confidence level of 95%, a margin of error of 5%, an expected proportion of 30% and a non-response rate of 30%, resulting in 460 surveys needed to guarantee 322 effective surveys. The participation of the different regions will be monitored so that the final sample distribution is proportional to the population size of each region, ensuring a balanced and representative international analysis. To this effect, a continuous monitoring system will be implemented throughout the data collection phase to oversee sample distribution in real-time. Should any variations or low response rates be identified within a specific professional group or geographical region, communication and dissemination efforts will be redeployed. This strategy aims to mitigate potential selection biases and ensure the overall robustness and representativeness of the data.

Participants will be healthcare professionals in nursing, medicine, and physical therapy, for whom the following inclusion criteria will be considered: (a) be enrolled in HE at a public or private educational institution in Health sciences or (b) be active healthcare professionals in Primary Care, Specialized Care or private centers and (c) being a native speaker of the language of data collection at each university. The exclusion criteria are (a) having special educational needs as the questionnaire will not be adapted and (b) not practicing or aid in pediatrics units (Primary or Specialized Care). Nevertheless, it is anticipated that future versions of the questionnaire will incorporate accessibility adaptations and features to enable the participation of individuals with diverse educational needs, thereby fostering a broader and more inclusive humanization approach.

A publicly accessible report on the results and the international baseline situation in this field will be disseminated to identify strengths and weaknesses. After launching the questionnaire and obtaining a representative international sample, a comprehensive quantitative analysis will be conducted, including analyses by gender, age group, continent, profession, and area of study for each item. In addition, segmentation and comparative analyses will be performed using Chi-square tests to evaluate statistical associations and to reveal the complex global landscape, including corresponding strengths and weaknesses, regarding the humanization of pediatric pain care and its non-pharmacological approach among healthcare professionals worldwide.

Based on the analysis carried out after the launch and the collection of international data on the health training needs of healthcare professionals, a database of training material will be developed to carry out three training courses focused on expanding the theoretical and practical knowledge of professionals and trainers, so that they can perpetuate this knowledge by training their peers. The material produced must comply with the criteria set out in the following work package in terms of content and format characteristics, so that it is suitable for face-to-face training and can be shared via the academic platform to spread knowledge and facilitate access, ensuring equal acquisition of knowledge.

**Figure 3:**
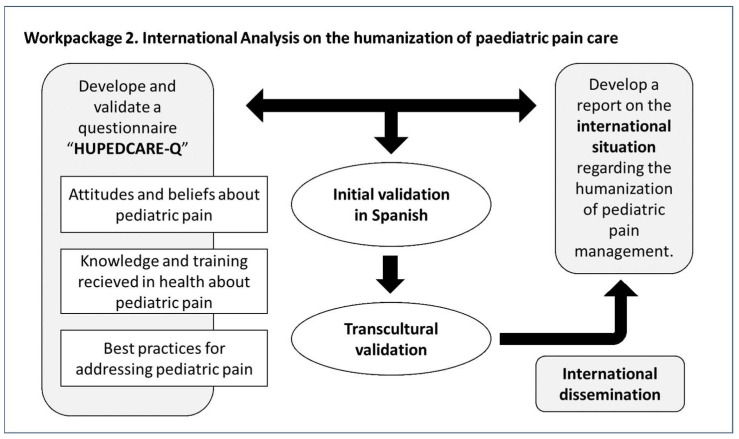
Process of the “International analysis of the humanization of pediatric pain treatment”. Source: authors’ own elaboration.

All the process is shown in Figure 3, illustrating the different steps that will be carried out, from questionnaire validation to international dissemination, in order to analyze the global situation.

### 2.4. Workpackage 3: PEDCARE Academic Web Platform

Open access, multi-device and multilingual web portal will be developed that will allow two main services. Firstly, it will be a learning management, monitoring and evaluation system (Learning Management System, or LMS) for educational courses or training programs in the humanization of pediatric pain care (Quilla Díaz et al., 2021). As an innovative element, it will integrate the use of interactive material and assistance through chatbots that effectively guide users within the environment. Each training course within the platform will have interactive self-assessment activities, texts, educational and interactive videos and discussion forums to promote the exchange of knowledge. But it will also act as a Social Networking Site (SNS) connecting experts in pediatrics to promote the exchange of knowledge and experiences and to generate collaborations between registered users on platform (Cajo et al., 2018). In addition, it is anticipated that future iterations of the platform will include accessibility tools to support diversity. The sustainability of the PEDCARE platform is ensured through the formal commitment associated with European public funding to maintain the platform and its website in operation for a minimum period of five years. 

Training the trainers will be conducted to provide the necessary knowledge to the project professionals to implement and create new training programs within the PEDCARE platform. In order to ensure the usability, accessibility, and suitability of the PEDCARE platform across different levels of digital literacy and cultural contexts, a pilot study will be conducted prior to its full deployment, scheduled for the first quarter of 2026. This phase will involve at least two members from each partner university, who will participate in the train-the-trainers program and will be deliberately selected to ensure diversity in cultural backgrounds, educational profiles, and levels of technological and digital competence. This pilot phase will allow for a systematic evaluation of user–platform interaction, comprehension of interactive materials, use of chatbots, and content management processes, thereby enabling iterative refinements before the full implementation of the system.

### 2.5. Workpackage 4: Capacity Building in the Field of Pediatric Management Care

This WP is divided into three training modules, each of which will focus on a specific topic related to childhood pain and will be delivered in stages at three universities participating in the HUPEDCARE project. The training sessions’ primary outcome is the improvement in theoretical knowledge and clinical competencies, evaluated via pre- and post-training assessments. Secondary outcomes include participants’ self-efficacy in humanistic care, satisfaction with the ‘PEDCARE’ platform, and the institutional replicability of the modules to ensure model scalability. There will be three training sessions: (1) Physiology and psychological aspects of pain; (2) Approaching pain in healthcare and community settings; (3) Family coping strategies for perceiving and responding to pediatric pain, all with a humanistic and comprehensive approach. Each trainer training course will have a specific target audience and will be held at different universities in Europe and America. The three training courses will initially be face-to-face, although it is envisaged that they will be replicated at more universities throughout the project period and may also be broadcast or hosted on the project’s own academic platform “PEDCARE”. The details of the training courses are outlined below:

#### 2.5.1. Capacity-Building Workshop for Trainers 1 “Physiology and Psychological Aspects of Pain”

This training will include mandatory content on the physiology of pain and its psychological aspects, with the aim of understanding pain in its entirety and from all perspectives of the biopsychosocial model. The additional training content will include key aspects such as sociocultural attitudes and beliefs about pediatric pain, the neurophysiological and psychological foundations of pain, and a multidimensional understanding that considers physical, emotional, social, and cultural factors. It will also address the long-term consequences of untreated pain in child development and introduce development-centered care approaches, including the NIDCAP model and other relevant frameworks based on local practices.

The target audience for this training will be academic professionals and HE students, with the aim of starting the training series at a higher level. To this end, the inclusion criteria will be as follows: students in the Health Sciences field who have knowledge of, or are currently studying, topics related to maternal and pediatric health; theoretical and practical instructors involved in the education of Health Sciences students in the areas of maternal, child, and pediatric health, both in classroom and clinical settings; and individuals who demonstrate an interest in participating in the activity.

The training aims to empower participants to promote the humanization of care in pediatric pain management. It seeks to foster cultural inclusion by identifying beliefs about pediatric pain, recognize its specific characteristics from the prenatal stage onward, define pain from a multidimensional perspective, and introduce principles of development-centered care. The first training session will be held in Turkey in October 2025. It will be a face-to-face training session in which all project partners will meet, share, and acquire new knowledge.

#### 2.5.2. Capacity-Building Workshop for Trainers 2 “Approaching Pain in Healthcare and Community Settings”

This training will include mandatory content on addressing pediatric pain in healthcare and community settings, with the aim of expanding knowledge and providing different tools, including the use of technology and new medical advances, to improve child healthcare in a humane way. The additional contents of this training include a description of the environment in neonatal intensive care units (NICUs) and the causes of discomfort and pain in neonatal patients in this context, the recognition of pain as the fifth vital sign, the assessment and evaluation of pain using validated instruments and scales, as well as an overview of both non-pharmacological and pharmacological interventions in the management of pediatric pain. The purpose of this training is to enable professionals to describe the most evidence-based pharmacological and non-pharmacological measures and to apply pain recognition and assessment scales through practical case studies and real-life simulations.

The second training course will be held at the University of Sao Paulo in Brazil in May 2026, coinciding with the first international congress of the HUPEDCARE project (an event that forms part of the workpackage 5). The inclusion criteria for participants in the first training are: being a health professional or health student, being an operational or educational support assistant, and demonstrating interest in participating in the activity.

#### 2.5.3. Capacity-Building Workshop for Trainers 3 “Family Coping Strategies for Perceiving and Responding to Pediatric Pain”

The latest mandatory in-person training will cover the use of family strategies for pediatric pain perception and response, in which social and healthcare professionals from rural and urban settings (daycare centers, rural and urban hospitals, urban and rural health centers, specialized centers, etc.) will be trained in the humanization of pediatric pain care. By expanding the tools available to parents or legal guardians of pediatric patients, it is possible to delve deeper into how to improve parents’ self-care in critical situations in which their children are experiencing pain and how to treat them. Additional training will emphasize partnering with parents in pediatric pain care, applying family-centered assessment models, and understanding pain’s multidimensional nature. It will cover age-appropriate pain scales, family coping strategies, non-pharmacological relief methods, and safe management of prescribed medications in both rural and urban contexts.

The inclusion criteria for this training will include families with children and/or adolescents living in rural or urban communities, who express an interest in participating in the activity. In addition, professionals from rural and urban socio-health settings who wish to expand their knowledge to later share it within healthcare environments—such as clinics and community services—will also be welcome, with the goal of enabling broader family outreach and education on this topic. This workshop will be held in November 2026 by the University of Évora in Portugal.

**Figure 4:**
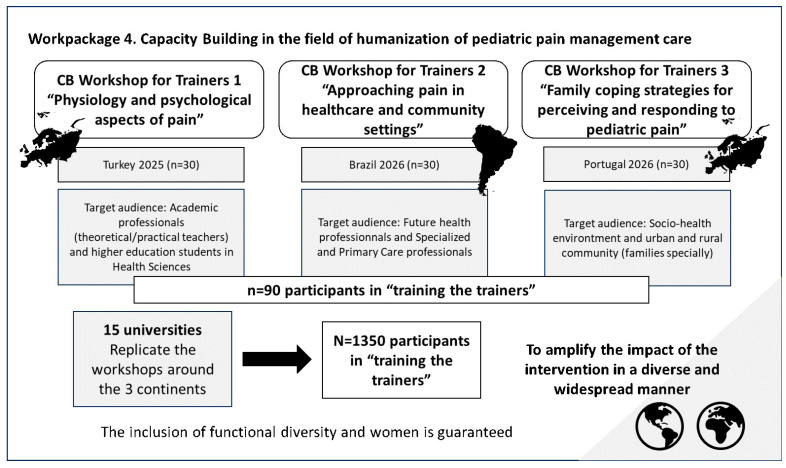
Procedure for conducting “training-the-trainers” sessions for work package 4. Source: authors’ own elaboration.

Procedure: This range is intended to ensure that all attendees can ask questions, receive adequate responses, and remain fully engaged throughout the activity. The total sample of participants in the initial training sessions will comprise a maximum of 90 individuals (assuming 30 participants per session). This sample will include individuals from various regions of each country, thereby ensuring that their socio-cultural characteristics are representative of the participating universities. Each session will last 180 min, during which a pre-intervention questionnaire (the validated HUPEDCARE questionnaire) will be administered. The training itself will consist of theoretical and practical activities, group discussions, and case study analysis. Upon conclusion of each session, two additional instruments will be administered: a post-intervention questionnaire and a validated satisfaction survey assessing the quality of the instruction, the training environment, and the performance of the teaching staff (Soto-Fernández et al., 2024). The procedure for training the trainers is shown in Figure 4.

Pre- and post-intervention scores will be compared using paired-sample t-tests or Wilcoxon signed-rank tests to evaluate statistical significance (Field, 2013). While this protocol focuses on immediate educational impact, medium- and long-term outcomes—such as knowledge retention and clinical implementation—are acknowledged as key objectives for future longitudinal research (Kirkpatrick & Kirkpatrick, 2006).

These training sessions will be replicated after the completion of the three official workshops held in Turkey, Portugal, and Brazil. Consequently, all participating universities will be expected to carry out additional training sessions based on the material developed during the initial workshops. The total number of people receiving training, considering only those recruited by the 15 universities participating in the project and who will replicate the three training courses in their regions, is expected to reach approximately 1350 participants. To ensure access to these workshops, participation will be facilitated for disadvantaged groups, such as those with functional diversity, to bring this knowledge to all interested parties who meet the inclusion criteria (Silverman et al., 2010). In addition, in line with SDG 5, “Gender Equality,” the participation of women in these workshops will be considered to enable the inclusion of women in HE in the most disadvantaged areas (Orr et al., 2024; Feltes et al., 2019).

The total number of indirect beneficiaries of these training sessions cannot be accurately calculated, as the nature of the activity is that of training the trainers. The “training the trainers” workshops are aimed at training people in the humanization of pediatric pain care, so that they can pass on, teach, and raise awareness among others about the application of the strategies and skills acquired.

In this sense, this educational process serves to consolidate learning and promote a human approach to pediatric care and the humanization of pain care (Orr et al., 2024; Feltes et al., 2019; Nexø et al., 2024). Participants in these sessions will subsequently pass on the knowledge they have acquired by training other educators and professionals within their environment. As a result, the knowledge disseminated through the training workshops will be extended to people from diverse backgrounds and health disciplines, thereby amplifying the impact of the intervention in a diverse and widespread manner.

### 2.6. Workpackage 5: Promotion of HUPEDCARE and Exploitation of Results

To promote the work of the HUPEDCARE project and expand its network while creating future research opportunities focused on the humanized approach to pediatric pain management, seminars and specialized training courses will be conducted through the platform. Additionally, efforts will be made to maximize the dissemination and utilization of the project’s results and interventions by organizing two international conferences. These events will showcase the project’s work and facilitate the establishment of a network of experts who can participate in and collaborate with the project, thereby ensuring the sustainability of HUPEDCARE’s mission over time, reaching a broader audience, and fostering innovation and new knowledge in humanized pediatric pain treatment options.

## 3. Discussion

The HUPEDCARE project is a transformative and highly relevant initiative that addresses a critical and traditionally neglected area worldwide: the humanization of pediatric pain care (Mozo del Castillo et al., 2022; Tripodi et al., 2017; Bernadá & Notejane, 2022). This international multicenter study presents the fusion of higher education, the humanization of healthcare, and technological-digital innovation, becoming a potential catalyst for global change in healthcare training and service delivery (Van Eck et al., 2021; Ryan et al., 2022; Ioannou & Avraamides, 2020). Aligned with SDGs 3 and 5, the focus of the project and its health training favors the inclusion of all people, regardless of gender or functionality, resulting in a strategic proposal due to its commitment to improving the health of a vulnerable group such as children and adolescents (Sanhueza et al., 2022; Orr et al., 2024).

However, the implementation of such an ambitious international multicenter study is not without significant limitations and risks. A primary challenge lies in the heterogeneity of sociocultural and educational contexts across the 15 participating universities. While the HUPEDCARE-Q questionnaire is validated in multiple languages to standardize competency assessment (Li et al., 2022; Davis et al., 2025), the baseline level of pediatric pain training and the digital literacy of students and professionals vary considerably between regions. In contexts like Mozambique or Peru, where resources may be scarce (Okoroafor et al., 2022; Scheffer et al., 2025), the reliance on online study platforms introduces a risk of a digital divide, potentially limiting the reach of the intervention if local technological infrastructure is insufficient.

Furthermore, while the “training the trainers” model (Quilla Díaz et al., 2021; Halabieh et al., 2022) aims for sustainability, there is an inherent challenge in ensuring that a standardized curriculum remains culturally sensitive without losing its core evidence-based structure. Transnational collaboration provides fertile ground for sharing knowledge, but it also requires a nuanced approach to overcome potential resistance to non-pharmacological interventions in regions where traditional medical models are deeply entrenched (Yousafzai et al., 2014; Rowe et al., 2018; Frenk et al., 2010).

Despite these challenges, the project’s emphasis on family-centered care and sociocultural determinants represents a necessary paradigm shift (García-Valdivieso et al., 2025; Gómez-Cantarino et al., 2020). By explicitly addressing these contextual disparities, HUPEDCARE seeks not only to deliver a universal tool but to adapt its humanized care model to the specific emerging global health needs of each participating nation (Stroud et al., 2019; Kruk et al., 2018).

## 4. Conclusions

In conclusion, despite challenges in accessing specialized pediatric education and technological infrastructure, the transnational scope of this project seeks to foster collaborative capacity-building among healthcare students, educators, and professionals. Thus, the multicentric and culturally connected nature of the study is designed to enhance training and potentially contribute to reducing health disparities and strengthening local healthcare systems. Rather than predetermining outcomes, this project aims to provide a framework for achieving more equitable health care for children in underrepresented regions. As a study protocol, it is expected to lay the groundwork for future research and evidence-based results. Therefore, the next critical step will be to analyze and evaluate the long-term impact of the project on healthcare outcomes and the experience of pediatric patients.

## Data Availability

No new data were created or analyzed in this study.

## Abbreviations

CBHE	Capacity Building in higher education
CEIS	Comité de Ética en Investigación Social
EAECA	European Education and Culture Executive Agency
HE	Higher Education
HUPEDCARE	Higher Education as a driver for the Humanization of Pediatric Pain Care
HUPEDCARE-Q	Humanization of Pediatric Care in Pain Management with a Non-Pharmacological Approach Questionnaire
IASP	International Association for the Study of Pain
ICT	Information and Communication Technologies
LSM	Learning Management System
SDG	Sustainable Development Goals
SNS	Social Networking Site
WHO	World Health Organization
WP	Workpackage
